# Decrypting the *Polyporus dictyopus* complex: Recovery of *Atroporus* Ryvarden and segregation of *Neodictyopus* gen. nov. (Polyporales, Basidiomyocta)

**DOI:** 10.1371/journal.pone.0186183

**Published:** 2017-10-19

**Authors:** Melissa Palacio, Gerardo Lucio Robledo, Mateus Arduvino Reck, Emanuel Grassi, Aristóteles Góes-Neto, Elisandro Ricardo Drechsler-Santos

**Affiliations:** 1 Programa de Pós-Graduação em Biologia de Fungos, Algas e Plantas, Departamento de Botânica, Universidade Federal de Santa Catarina, Florianópolis, Santa Catarina, Brasil; 2 Instituto Multidisciplinario de Biología Vegetal, Universidad Nacional de Córdoba, Córdoba, Argentina; 3 Instituto Misionero de Biodiversidad (IMiBio), Puerto Iguazú - Misiones–Argentina; 4 Molecular and Computational Biology of Fungi Laboratory, Department of Microbiology, Institute of Biological Sciences, Federal University of Minas Gerais, Belo Horizonte, Minas Gerais, Brazil; Friedrich Schiller University, GERMANY

## Abstract

*Polyporus dictyopus*, with a large number of heterotypic synonyms, has been traditionally considered a species complex, characterized by wide morphological variation and geographic distribution. Thus, neotropical specimens previously identified as *P*. *dictyopus* from Amazonia, Cerrado and Atlantic Forest biomes were studied based on detailed macro- and micromorphological examination and phylogenetic analyses, using distinct ribosomal and protein-coding genomic regions: the nuclear ribosomal internal transcribed spacer (nrITS), nuclear ribosomal large subunit (nrLSU), and RNA polymerase II second subunit (RPB2). Two unrelated generic lineages, each one represented by different species, are reported: *Atroporus* is recovered and re-circumscribed to include *A*. *diabolicus* and *A*. *rufoatratus* comb. nov.; *Neodictyopus* gen. nov. is proposed to accommodate *N*. *dictyopus* comb. nov. and two new species, *N*. *atlanticae* and *N*. *gugliottae*. Our study showed that at least five distinct species were hidden under the name *P*. *dictyopus*. Detailed descriptions, pictures, illustrations, and a key are provided for *Atroporus* and *Neodictyopus* species.

## Introduction

· *Polyporus* P. Micheli ex Adans has been traditionally characterized by its stipitate basidiomata, poroid hymenophore, dimitic hyphal system with skeletal-binding hyphae, and cylindrical to ellipsoid basidiospores. In this wide sense, it has been considered with a wide global distribution and encompassing other names as congeneric synonyms [1–6]. *Polyporus* was divided into six non-taxonomic morphological groups: “Polyporus”, “Favolus”, “Melanopus”, “Polyporellus”, “Admirabilis”, and “Dendropolyporus” due to its macromorphological heterogeneity and also for practical use [1].

Recent phylogenetic analyses revealed that these *Polyporus* morphological groups do not comprise entirely independent monophyletic lineages [7]. Additionally, *Polyporus* has been considered polyphyletic [7–11]. Based on phylogenetic and morphological analyses of the “Favolus group”, the name *Favolus* Fr. was recovered and re-circumscribed for the nesting species into the clade A, and *Neofavolus* Sotome & T. Hatt. was segregated as an independent genus for the clade B [12]. On the other hand, “Melanopus group”, which is characterized by its coriaceous basidiomata with a black cuticle in the stipe [1], corresponds to an artificial group. However, several species close to *Polyporus melanopus* (Pers.) Fr. remain grouped and defined as the "melanopus clade" [10]. This clade has received a taxonomical approach and has been recently accommodated as *Picipes* Zmitr. & Kovalenko [13], and *Cladomeris* and they are characterized by its dark pileus (smoke gray to chesnut or deep brown) and black stipe [13,14].

These studies also showed that several other taxa, such as *Polyporus leprieurii* Mont., *P*. *guianensis* Mont. and *P*. *dictyopus* Mont., preliminarily considered to be in the "Melanopus group", are not phylogenetically related and outside *Picipes*, in another clade named "squamosus", which is characterized by a black cuticle at the base or in the entire stipe [14]. *Polyporus guianensis* and *P*. *leprieurii* have pale brown tan to beige pilei, a morphological character that distinguishes them from *Picipes*; however, *P*. *dictyopus* shares the same general features as those described as diagnostic for *Picipes*. *Polyporus dictyopus* was originally described based on a collection from the Juan Fernandez archipelago, near the coast of Chile, and considered as a species complex, based on morphology and mating type data [1,6]. The current concept of *P*. *dictyopus* comprises a wide variation of the pilear surface color (chestnut to purplish black), stipe insertion (lateral to centrally stipitate), and basidiospore sizes and shapes (ellipsoid to cylindrical). *P*. *dictyopus* has a wide global distribution and a large number of heterotypic synonyms (with at least 16 known from tropical and subtropical America) due to this wide range in its morphological characters [1,15].

Two of those heterotypic synonyms, *Polyporus diabolicus* Berk. and *P*. *infernalis* Berk., were previously accommodated in *Atroporus* Ryvarden due a particular feature: hymenial cystidia with protuberances and a “sharply pointed apex” [16]. Later, *Atroporus* cystidia were reinterpreted as modified binding hyphae [17], and the synonymization of *Atroporus* in *Polyporus* was proposed, making *P*. *diabolicus* and *P*. *infernalis* synonymys of *P*. *dictyopus* [1]. This criterium was assumed in later studies [2,6,15,18,19].

The morphological heterogeneity and the global distribution of *P*. *dictyopus* strongly suggest that there is a hidden and underestimated taxonomic diversity under this species concept. In our study, we aim to performed a detailed morphological and molecular analyses of the specimens previously identified as *P*. *dictyopus* from the neotropics in order to access the diversity of species supported by morphological, phylogenetic, and ecological (distribution, ecosystem preference) evidence.

## Materials and methods

### Collections and morphological studies

For species distribution, we used the Neotropical regionalization proposed by Morrone [20], for substrate we used the terminology from Kruys & Jonsson [21]; where fine woody debris (FWD) have a diameter of 5–9 cm and coarse woody debris (CWD, ≥10 cm). Specimens were collected in the Boreal Brazilian, Cerrado, Paraná, and Southeastern Amazonian domains, in the Brazilian and Chacoan subregions in the States of Amazonas, Bahia, Santa Catarina, São Paulo (Brazil), and Province of Misiones (Argentina). Voucher specimens were deposited in FLOR, CORD and BAFC. All necessary permits for field collections were obtained. This study does not involve endangered or protected species. We also examined several reference specimens, including type specimens of NY and BPI (herbarium acronyms follow Thiers [22]). Color descriptions were given according to Munsell [23]. Microscopic observations were made from freehand cross sections of dried materials mounted in Melzer’s reagent, 5% KOH and/or, 1% phloxine, lactophenol, cresyl blue or cotton blue (CB). To observe the hyphal system, we followed the technique described by Decock et al. [24]. Basidiospore measurements were made in Melzer’s reagent (n = 40). The meanings of abbreviations are as follow: IKI– = inamyloid and indextrinoid, CB+/– = cyanophilous/acyanophilous, ave = arithmetic mean and Q = the ratio of length/width of basidiospores. In presenting the size range of several microscopic elements, 5% of the measurements at each end of the range are given in parenthesis, when relevant. We followed Stalpers [25] and the Stalpers database (http://www.cbs.knaw.nl/russulales/) for the basidiospore shape terminology.

### DNA extraction, PCR amplification and sequencing

DNA was extracted from dried specimens using the Doyle & Doyle [26] protocol adapted by Góes-Neto et al. [27]. The partial regions of (i) the nuclear ribosomal internal transcribed spacer (nrITS), (ii) nuclear ribosomal large subunit (nrLSU), and (iii) RNA polymerase II second subunit (RPB2) were amplified by Polymerase Chain Reaction. The primer pairs used for amplification were ITS8F-ITS6R [28], LR0R-LR7 [29] and fRPB2-5F and bRPB2-7.1R [30,31], respectively. The parameters of amplification for each region were followed as described by Dentinger et al. [28], Vilgalys & Hester [29], and Frøslev et al. [30], and Matheny [31], respectively. The PCR products were purified with PEG 20% [Poly(ethylene glycol) 8,000 plus NaCl 2.5M] and then were sequenced with BigDye Terminator 3.1 Cycle Sequencing Kit following manufacturer procedures, using the same primer pairs for nrITS; LR0R - LR5 for nrLSU, and fRPB2-5F, bRPB2-6F and bRPB2-7.1R for RPB2 at FIOCRUZ-MG (Brazil) as part of the FungiBrBol project (www.brbol.org). The sequences and chromatograms were manually checked and edited with Geneious 6.1.8 [32]. Sequences newly generated in this study were submitted to GenBank (Table 1).

**Table 1:** List of species, collections, and GenBank accession numbers for the nrITS, nrLSU, and RPB2 sequences used in the phylogenetic analyses.

Species	Strain/Specimen No.	Locality	GenBank accesion No.		
			nrITS	nrLSU	RPB2
*Atroporus diabolicus* (Berk.) Ryvarden	DS1266	Amazonas, Brazil	KY631757	KY631768	-
*A*. *rufoatratus* (Berk.) Palacio, Reck & Robledo	DS1311	Santa Catarina, Brazil	KY631758	KY631769	-
	DS816	Santa Catarina, Brazil	KY631759	KY631770	KY744947
	MP153	Santa Catarina, Brazil	KY631760	KY631771	-
*Datronia mollis* (Sommerf.) Donk	Dai 11253	China	JX559258	JX559289	JX559306
	RLG 6304	USA	JN165002	JN164791	JN164872
*D*. *stereoides* (Fr.) Ryvarden	Holonen	Finland	KC415179	KC415196	KC415202
*Neodictyopus atlanticae* Palacio, Robledo & Drechsler-Santos	DS1285	Santa Catarina, Brazil	KY631762	KY631773	KY744949
	DS1286	Santa Catarina, Brazil	KY631763	KY631774	KY744950
	FB351	Santa Catarina, Brazil	KY631764	KY631775	KY744951
*N*. *gugliottae* Palacio, Grassi & Robledo	GAS622	Sao Paulo, Brazil	KY631761	KY631772	KY744948
	CI110	Misiones, Argentina	KY765022	KY765023	-
*N*. *dictyopus* (Mont.) Palacio, Robledo & Drechsler-Santos	GAS60	Mato Grosso, Brazil	KY631765	KY631776	-
	GAS272	Mato Grosso, Brazil	KY631766	KY631777	KY744952
	GAS281	Mato Grosso, Brazil	KY631767	KY631778	KY744953
*Echinochaete russiceps* (Berk. & Broome) D.A. Reid	WD674	Japan	AB462310	AB368065	AB368123
*Favolus brasiliensis* (Fr.) Fr.	INPA241452	Brazil	AB735977	AB735953	-
	TENN10242	Costa Rica	AB735976	AB368097	-
*F*. *emerici* (Berk. ex Cooke) Imazeki	WD2343	Japan	AB587626	AB368089	AB368146
	WD2379	Japan	AB587628	AB587619	AB368147
*F*. *roseus* Lloyd	PEN33	Malaysia	AB735975	AB368099	AB368156
*Mycobonia flava* (Sw.) Pat.	TENN59088	Argentina	AY513571	AJ487933	-
	TENN57579	Costa Rica	AY513570	AJ487934	-
*Neofavolus alveolaris* (DC.) Sotome & T. Hatt.	WD2340	Japan	AB735970	AB368077	AB368135
	WD2358	Japan	AB587624	AB368079	AB368136
*N*. *cremeoalbidus* Sotome & T. Hatt.	TUMH 50009	Japan	AB735957	AB735980	-
*N*. *mikawai* (Lloyd) Sotome & T. Hatt	TUMH 50005	Japan	AB735964	AB735944	-
*Neodatronia sinensis* B.K. Cui, Hai J. Li & Y.C. Dai	Cui 9434	China	JX559271	JX559282	JX559319
	Dai 11921	China	JX559272	JX559283	JX559320
*Picipes badius* (*Pers*.*) Zmitr*. *& Kovalenko*	WD2341	Japan	AB587625	AB368083	AB368140
*P*. *conifericola* (H.J. Xue & L.W. Zhou) J.L. Zhou & B.K. Cui	WD1839	Japan	AB587634	AB368101	-
*P*. *melanopus* (Pers.) Fr.	MJ 372–93	Czech	KC572026	KC572065	-
	H 6003449	Finland	JQ964422	KC572064	-
*Polyporus dictyopus* Mont.	TENN 59385	Belize	AF516561	AJ487945	-
	WD1845	Japan	-	AB368085	AB368142
	WD2342	Japan	-	AB368086	AB368143
	WD2345	Japan	-	AB368087	AB368144
*P*. *leprieurii* Mont.	TENN58597	Costa Rica	AF516567	AJ487949	AB368150
*P*. *squamosus* (Huds.) Fr.
	AFTOL ID-704	USA	DQ267123	AY629320	DQ408120
*P*. *tuberaster* (Jacq. ex Pers.) Fr.	WD2382	Japan	AB474086	AB368104	AB368161
*P*. *udus* Jungh.	WD1878	Japan	-	AB368108	AB368165
*P*. *umbellatus* (Pers.) Fr.	WD719	Japan	-	AB368109	AB368166
*P*. *varius* (Pers.) Fr.	WD619	Japan	AB587635	AB368110	AB368167
*Trametes hirsuta* (Wulfen) Lloyd	RLG5133T	USA	JN164941	JN164801	JN164854
*T*. *versicolor* (L.) Lloyd	FP135156sp	USA	JN164919	JN164809	JN164850

### Phylogenetic analyses

Two distinct datasets were constructed: the first based on three molecular markers (nrITS, nrLSU, and RPB2), and the second based on two (nrITS and nrLSU). The generated sequences, including related sequences downloaded from GenBank (Table 1), were aligned using Mafft v.7 [33], under the Q-INS-I strategy for nrITS and G-INS-i strategy for nrLSU and RPB2 for both datasets. The alignments were manually examined and adjusted with MEGA 6 [34].

We coded the nrITS and nrLSU indels present in the datasets as binary characters following the simple indel coding method [35], performed in the SeqState software [36]. An intron in RPB2 was separated and analyzed as a distinct partition. The first dataset was subdivided into nine partitions: ITS1, 5.8S, ITS2, nrLSU, RPB2 -1st, -2nd, -3rd codon positions, RPB2 intron, and ITS/LSU Indels; the second was subdivided into five partitions, excluding the partitions related to RPB2. The best-fit evolutionary model for every partition was selected using jModelTest v. 1.6 [37,38] following the Bayesian Information Criterion (BIC). The final alignments were deposited at TreeBASE (submission ID: S20479). To test the congruence of the distinct nucleotide partitions, we applied the Partition Homogeneity Test (PHT), as implemented in PAUP* [39]. Since this test does not show any incongruence among the partitions, we proceeded with the concatenated analyses. Two distinct analyses were performed for each dataset: Bayesian Inference (BI) and Maximum Likelihood (ML). Bayesian Inferences were conducted using MrBayes 3.2.6 as available in CIPRES Science Gateway 3.1 [40], and implemented with two independent runs, each one with four chains and starting from random trees. The runs performed 20.000.000 generations and trees were sampled every 1000^th^ generation. Twenty five percent of sampled trees were discarded as burn-in, while the remaining ones were used for calculating a 50% majority consensus tree and Bayesian Posterior Probabilities (BPP). To check the convergence and stability of the runs, the average standard deviation of split of frequencies (<0.01) was evaluated in Tracer v.1.6 [41], as well as the potential scale reduction factor (PSRF). ML trees were obtained using RAxML v.8.1.4 [42], in CIPRES science gateway [40]. The analysis first involved 100 ML searches, each one starting from one randomized stepwise addition parsimony tree, under a GTRGAMMA model, with no proportion of invariant sites and all other parameters estimated by the software. We provided a partition file to force RAxML software to search for a separate evolution model for each dataset. Bootstrap support values (BS) were obtained with multi-parametric bootstrapping replicates under the same model, allowing the program halts bootstrapping automatically by the autoMRE option. A node was considered to be strongly supported if it showed a BPP ≥ 0.95 and/or BS ≥ 90%, while moderate support was considered when BPP < 0.95 and/or BS < 90%. *Trametes hirsuta* (Wulfen) Lloyd and *Trametes versicolor* (L.) Lloyd were used as the outgroup based on previous studies [10,12].

### Nomenclature acts

The electronic version of this article in Portable Document Format (PDF) in a work with an ISSN or ISBN will represent a published work according to the International Code of Nomen- clature for algae, fungi, and plants, and hence the new names contained in the electronic publi- cation of a PLOS article are effectively published under that Code from the electronic edition alone, so there is no longer any need to provide printed copies.

In addition, new names contained in this work have been submitted to MycoBank from where they will be made available to the Global Names Index. The unique MycoBank number can be resolved and the associated information viewed through any standard web browser by appending the MycoBank number contained in this publication to the prefix http://www.mycobank.org/MB/. The online version of this work is archived and available from the following digital repositories: PubMed Central and LOCKSS.

## Results

### Phylogenetic analysis

**Fig 1:**
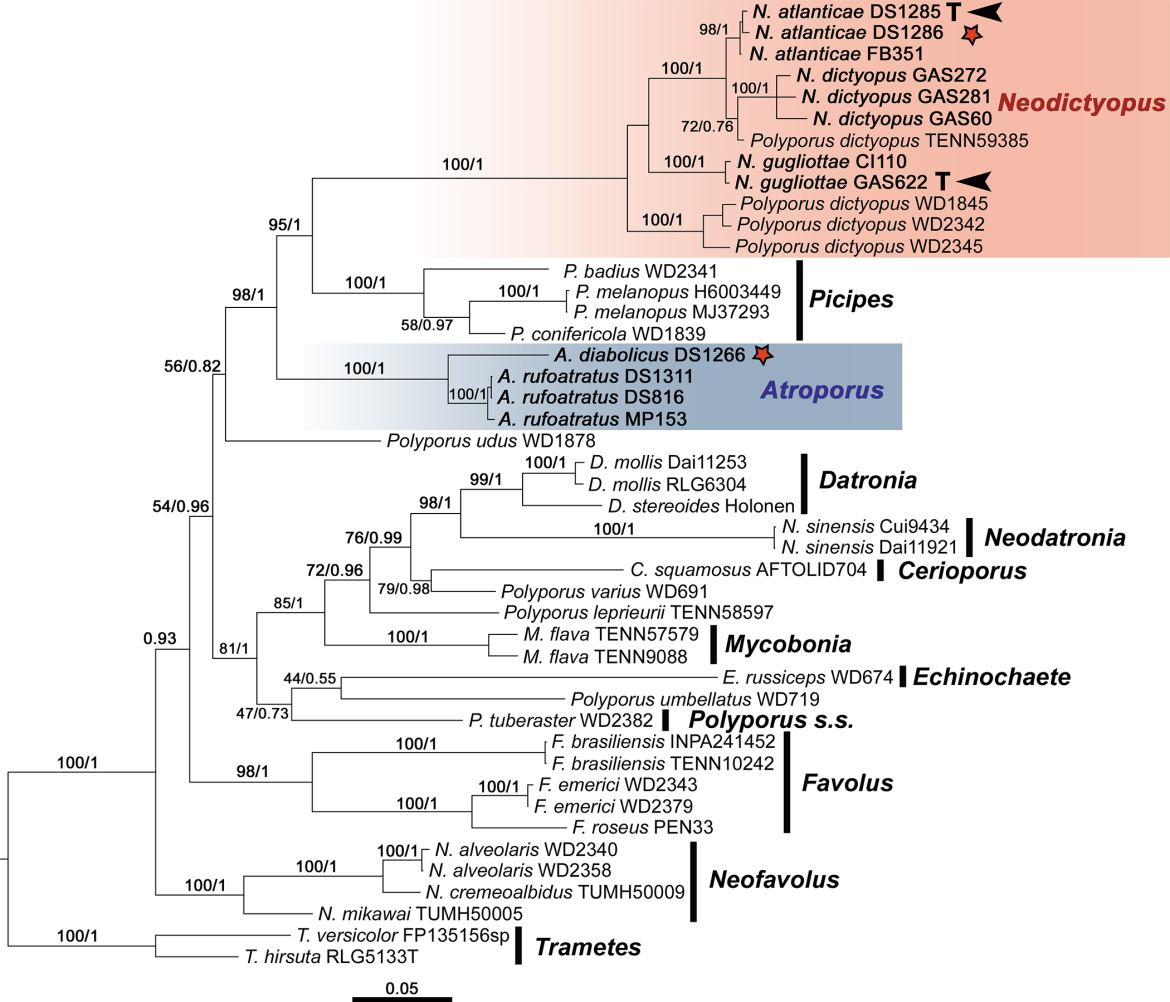
Phylogenetic relationships of members of the *Atroporus* and *Neodictyopus* clades inferred from ITS, nucLSU, and RPB2 sequences. Topology from Bayesian Inference analysis. Bootstrap support values (before the slash markers) and Bayesian posterior probabilities (after the slash markers) are indicated. Red asterisks indicate the type species of the genus.

A total of thirty one sequences were newly generated in this study (12 nrITS, 12 nrLSU, and seven RPB2). The first dataset included 45 specimens representing 28 putative species, including the genera *Datronia* Donk, *Echinochaete* Reid, *Favolus*, *Mycobonia* Pat., *Neodatronia* B.K. Cui, Hai J. Li & Y.C. Dai, *Polyporus*, and *Trametes* Fr. species. The final alignment (S1 File) consisted of 2521 bp, with 214 indels recoded, resulting in 2735 characters. The second dataset included 77 specimens representing 42 putative species, including *Datronia*, *Echinochaete*, *Favolus*, *Lentinus* Fr., *Mycobonia*, *Neodatronia*, *Polyporus*, *Pseudofavolus* Pat., and *Trametes* species. The final alignment of this second dataset (S2 File) consisted of 1482 bp, with 324 indels recoded, resulting in 1806 characters. The best-fit evolutionary model selected for each partition and related information was summarized in Table 2. The topology of the BI and ML of the first and second dataset analyses showed no inconsistency in any supported clades, as is shown in the BI tree (Fig 1). For the second dataset, the topology of the ML analyses has no inconsistency with the BI, and they recovered the same clades of the first dataset. The bootstrapping criteria of RAxML indicated 360 pseudo replicates as sufficient to access the internal branch support for the first dataset, and 204 for the second dataset.

**Table 2:** Summary of data sets of nrITS, nrLSU, and RPB2.

Properties	First Datasets								
	ITS1	5.8S	ITS2	nucLSU	RPB2 1st	RPB2 2nd	RPB2 3rd	RPB2 intron	Indels
**Model selected**	TIM2+G	K80+I	TrN+G	TIM2+I+G	TIM2+G	K80+G	TIM2+I+G	TrNef+G	F81-Like
**Likelihood score**	- 2570.1624	- 404.9665	- 2405.6239	- 3126.5433	- 1556.8426	- 1155.1076	- 5287.9108	- 1218.2834	–
**Base frequencies**									
**Freq. A =**	0.2000	Equal	0.2021	0.2588	0.2727	Equal	0.1258	Equal	–
**Freq. C =**	0.2134	Equal	0.2194	0.1899	0.2484	Equal	0.3391	Equal	–
**Freq. G =**	0.2417	Equal	0.2124	0.3029	0.3099	Equal	0.3181	Equal	–
**Freq. T =**	0.3450	Equal	0.3661	0.2484	0.1690	Equal	0.2171	Equal	–
**Proportion of invariable sites**	–	8.490	–	5.340	–	–	0.0320	–	–
**Gamma shape**	6.310	–	5.910	4.600	2.370	1.480	4.2260	2.4110	–

All phylogenetic analysis performed showed that specimens of *P*. *dictyopus* complex were grouped into two distinct, strongly supported clades, hereafter named “Atroporus clade” and “Neodictyopus clade”.

Within “Neodictyopus clade” (Fig 1) two clades can be observed. One grouped three species from the neotropics (BS = 77, BPP = 0.85): *P*. *dictyopus sp1* (BS = 98, BPP = 1), *P*. *dictyopus sp2* (BS = 100, BPP = 1), and *P*. *dictyopus sp3* (BS = 100, BPP = 1). The other clade grouped specimens from the paleotropics (subtropical Asia). Within the “Atroporus clade” (BS = 100, BPP = 1) two well supported species can be distinguished: *P*. *dictyopus* sp4 (BS = 100, BPP = 1) and *P*. *dictyopus* sp5.

*Polyporus tuberaster* (Jacq. ex Pers.) Fr., the type species of *Polyporus*, and here representing a *Polyporus s*.*s*. concept, was placed in a moderately supported clade (BS = 1, BPP = 81) grouped with *Datronia*, *Neodatronia*, *Polyporus s*.*l*. (*P*. *leprieurii*, *P*. *squamosus*, *P*. *umbellatus*, *P*. *varius* (Pers.) Fr.), *Mycobonia* and *Echinochaete* species. *Datronia*, *Echinochaete*, *Favolus*, *Mycobonia*, *Neofavolus*, *Neodatronia*, and *Picipes* were each supported as monophyletic, and “squamosus clade” [14] was not recovered by our phylogenetic analyses.

Morphological analysis showed that “Atroporus” and “Neodictyopus clades” have distinct morphological characters that separate them from *Polyporus* and *Picipes* as distinct genera. We propose the reappraisal and emendation of the genus *Atroporus* for the former clade and, for the latter clade, we propose the name *Neodictyopus* gen. nov.; as well as their respective species described and illustrated below.

### Taxonomy

***Atroporus*** Ryvarden, Norw. Jl Bot. 20: 2 (1973), emend. Palacio, Robledo, Reck & Drechsler-Santos.

**Fig 2:**
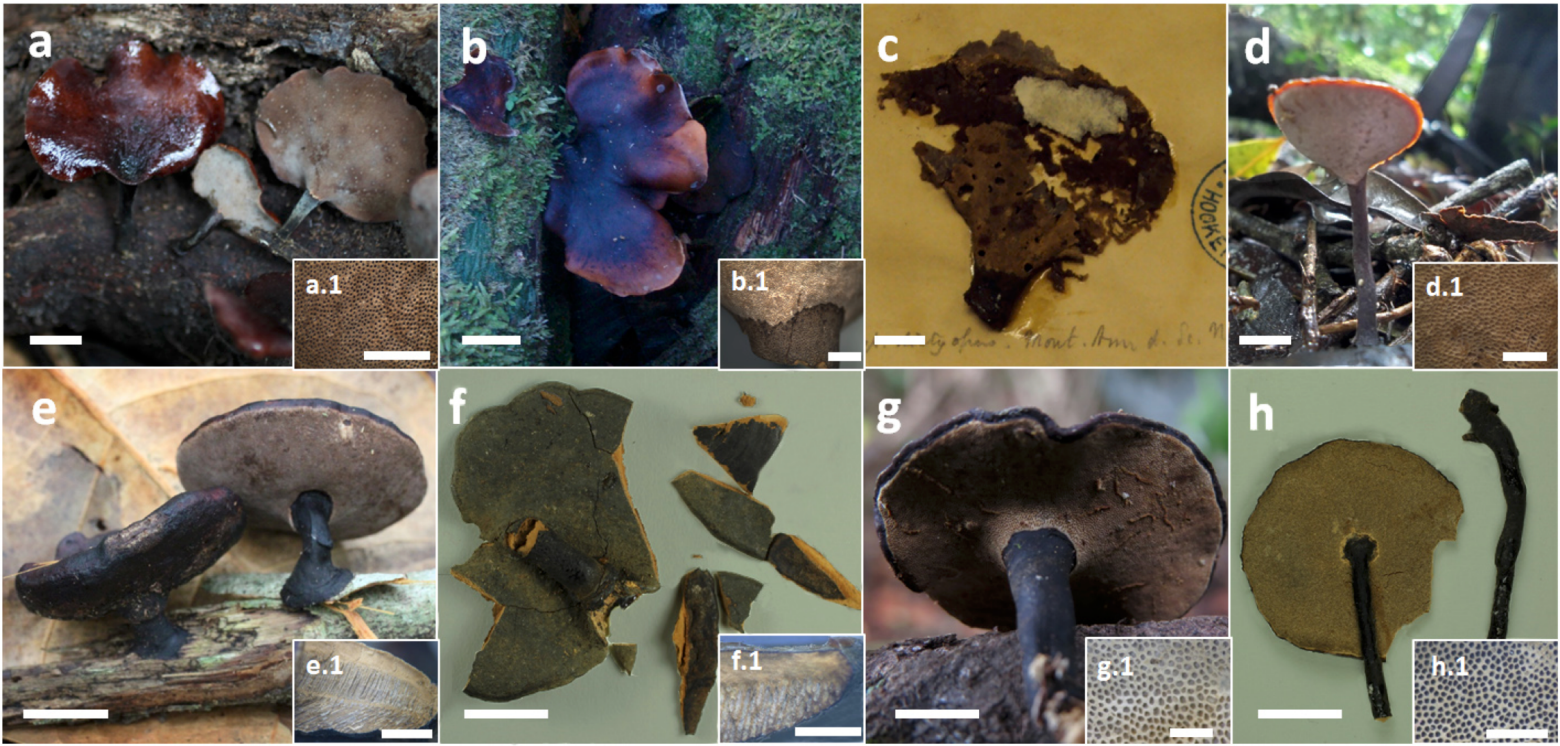
Basidiomata of *Neodictyopus* and *Atroporus* species. **a**. *N*. *atlanticae* (DS1284).**a1**. pores. **b**. *N*. *dictyopus* (GAS272). **b1**. pores and reticulated stipe. **c**. *N*. *dictyopus* type (Bertero 1683). **d**. *N*. *gugliottae* (GAS622). **d1**. pores. **e**. *A*. *diabolicus* (DS1266). **e1**. context and tubes. **f**. *A*. *diabolicus* type (NY 730627). **f1**. context and tubes. **g**. *A*. *rufoatratus* (LDA138). **g1**. pores. **h**. *A*. *rufoatratus* type (NY 730938). **h1**. pores. Scale bar: a, b, d-h = 1cm; c = 2 cm; a1-f1 = 2 mm; g1, h1 = 1 mm.

**Fig 3:**
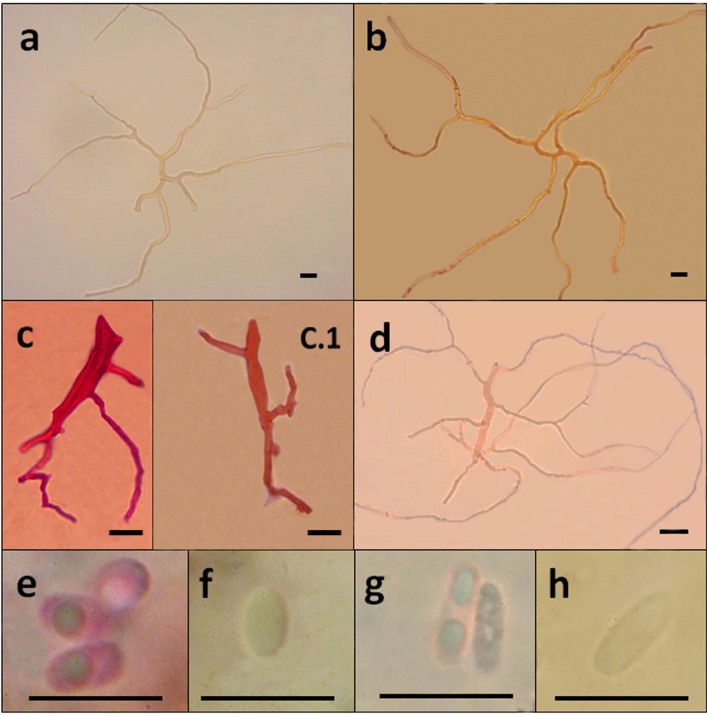
Comparison of microscopical features of *Atroporus* and *Neodictyopus*. Photos of: **a**. context hyphae of *A*. *diabolicus* (DS1266). **b**. context hyphae of *N*. *atlanticae* (DS1284). **c**. tramal hyphae *A*. *diabolicus* (DS1266). **c.1** tramal hyphae *A*. *rufoatratus* (LDA138). **d**. tramal hyphae of *N*. *atlanticae* (DS1284). **e**. basidiospores of *A*. *diabolicus* (DS1266). **f**. basidiospores of *A*. *rufoatratus* (MP153). **g**. basidiospores of *N*. *gugliottae* (GAS622). **h**. basidiospores of *N*. *atlanticae* (FB351). Scale black bar = 1 μm.

**Fig 4:**
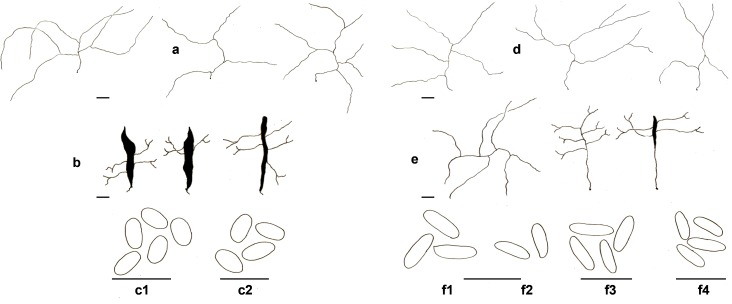
Comparison of microscopical features of *Atroporus* and *Neodictyopus*. Schematic drawings of: **a**. context hyphae of *A*. *diabolicus* (DS1266). **b**. tramal hyphae *A*. *diabolicus* (DS1266). **c1** basidiospores of *A*. *diabolicus* (DS1266). **c2** basidiospores of *A*. *rufoatratus* (MP153). **d.** context hyphae of *N*. *atlanticae* (DS1284). **e**. tramal hyphae of *N*. *atlanticae* (DS1284). Basidiospores of **f1**. *N*. *dictyopus* (GAS281), **f2**. (BPI US207664, type of *N*. *dictyopus*). **f3** *N*. *gugliottae* (GAS622). **f4** *N*. *atlanticae* (FB351). Scale black bar = 10 μm.

Basidiomata annual to biannual, centrally to eccentrically stipitate; pileus circular; pilear surface glabrous, radially striate to finely wrinkled, dark purplish red to blackish; margin sterile, with a black cuticle. Pores circular. Context homogenous, light brown. Stipe cylindrical, solid, bearing a black cuticle (Fig 2E, 2F, 2G and 2H). Hyphal system dimitic with generative and skeletal-binding hyphae; generative hyphae with clamp connections; skeletal-binding hyphae from the context and stipe usually dominating, arboriform, hyaline, IKI−; skeletal-binding hyphae in the trama of tubes dextrinoid, with differentiated and wide stalk, and sharply pointed apex. Basidia clavate, 4-sterigmate. Basidiospores narrowly ellipsoid to subcylindrical, thin-walled, smooth, hyaline, IKI–(Figs 3A, 3C, 3E, 3F, 4A, 4B, 4C1 and 4C2).

Type species. *Atroporus diabolicus* (Berk.) Ryvarden.

**Fig 5:**
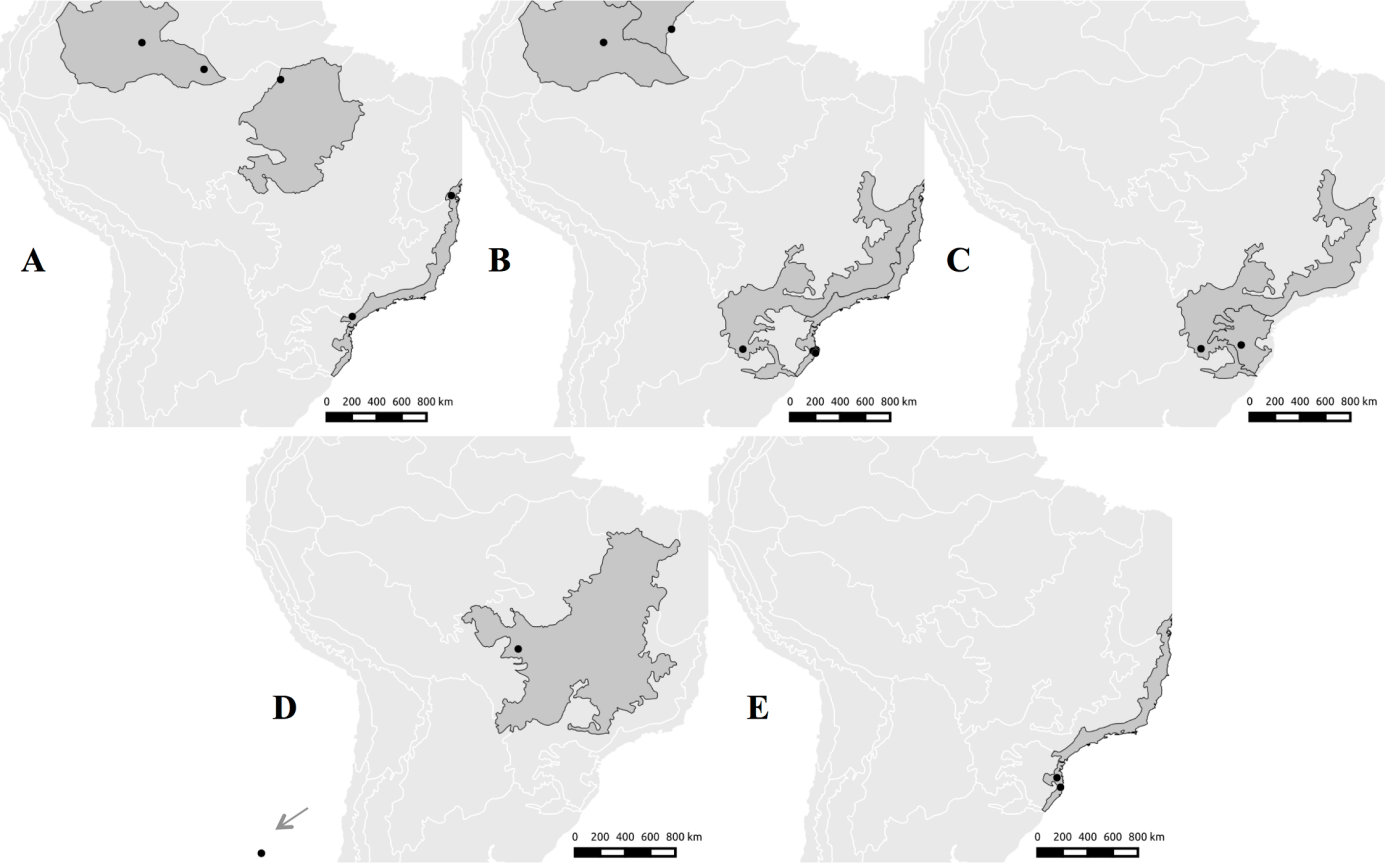
Potential geographic distribution of *Atroporus* and *Neodictyopus* species based on biogeographical regionalization of Morrone [20]. **A.** *A*. *diabolicus* (Atlantic, Imerí, and Xingu-Tapajós provinces). **B.** *A*. *rufoatratus* (Atlantic, Imerí, Pantepui, and Paraná Forest provinces). **C.** *N*. *gugliottae* (Araucaria and Paraná provinces). **D.** *N*. *dictyopus* (Cerrado province and Juan Fernandez archipelago). **E.** *N*. *atlanticae* (Atlantic province).

Remarks: Basidiospores descriptions and Melzer reagent reaction of the skeletal-binding hyphae are new diagnostic information for the genus. *Atroporus* resembles morphologically *Polyporus sensu lato* and *Echinochaete* Reid, however, the combination of narrowly ellipsoid to subcylindrical basidiospores, strongly dextrinoid skeletal-binding hyphae with a differentiated apex, and the black cuticle on the pileus are unique to the group *Atroporus*. All the species grow on dead wood, typically dead fallen branches of relatively thin diameter (up to 10 cm diam.) and cause white rot on the substrate. So far the genus is only known for the neotropics (Fig 5A and 5B).

***Atroporus diabolicus*** (Berk.) Ryvarden, Norw. Jl Bot. 20: 2 (1973) (Figs 2E, 2E1, 2F, 2F1, 3E, 4C1, 6A and 6B)

**Fig 6:**
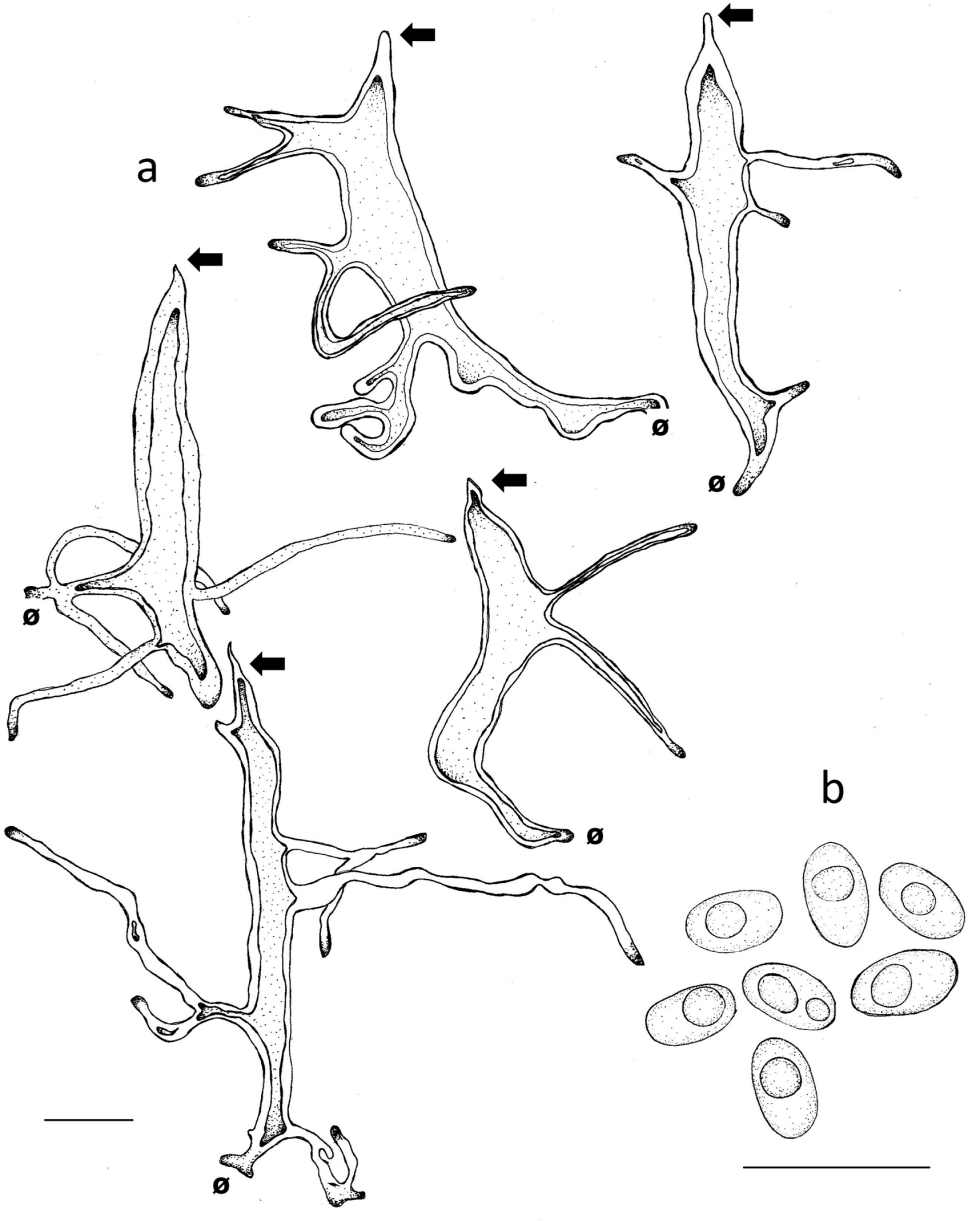
Microscopical features of *Atroporus diabolicus*. **a**. tramal hyphae (DS1266). **b**. ellipsoid basidiospores (GAS679). Ø = clamp scar. Left arrow: Pointed apex of the hyphae. Scale bars = 10 μm.

≡ *Polyporus diabolicus* Berk. Hooker's J. Bot. Kew Gard. Misc. 8: 174 (1856)!.

= *Polyporus vernicosus* Berk. Hooker's J. Bot. Kew Gard. Misc. 8: 175 (1856)!.

Basidiomata annual to biannual, central to eccentrically stipitate, solitary; pileus circular, up to 3.2 cm in diameter and 4 mm thick; pilear surface reddish black (10R2.5/1) to very dark red (2.5YR2.5/2), glabrous, radially striate to finely wrinkled; margin rounded/truncate, sterile, with a black cuticle. Pore surface light brown (7.5YR6/4) to dark brown (7.5YR3/2), in some specimens a black cuticle covering the surface; pores circular, regular, 5–8 per mm, 90–140(–150) μm (ave = 111.5 μm, n = 80/2); dissepiments entire, 30–100.5(–120) μm thick, (ave = 51.1 μm, n = 80/2). Tubes concolorous with pore surface, not stratified to stratified into 3 layers, up to 7 mm long each one. Context homogeneous, light brown (7.5YR6/4), 1.5 mm thick. Stipe cylindrical, solid, glabrous, longitudinally striate, bearing a black cuticle up to 3.2 cm long, up to 5 mm diam., with a robust appearance. Hyphal system dimitic with generative hyphae and skeletal-binding hyphae. Generative hyphae with clamps, hyaline, thin-walled, 2–3 μm thick, difficult to observe. Skeletal-binding hyphae of two types; arboriform type, present in stipe and context, up to 230 μm long, 2.5–4 μm wide, thick-walled, with a short unbranched stalk (17.5–48 μm), 4–6 branches with an alternating arrangement, and shortened as getting closer to the trama of the tubes, hyaline to yellowish in KOH and water, IKI- (Figs 3A and 4A). In the trama of the tubes are present the second type of hyphae (Figs 3C and 4B), skeletal-binding hyphae short (41–75 μm long) and "prickly" always with acute apex that is projected above hymenium, golden yellow in KOH and water, strongly dextrinoid changing to dark brown in Melzer reagent, thick-walled, just after the septa (3–5 μm wide) developing a stalk, that is considerably enlarged at the central portion (7–11 μm wide), between the middle portion and apical portion arise 2 to 6 branches (1–3 μm wide), stalk ending in an acute apex, as small spines, at angles 75°–90°, generally longer towards the base, which can reach up to 76 μm long, sometimes with dichotomous branches. Basidia clavate, 4-sterigmate, with a basal clamp, 19−22 × 6−8 μm. Cystidia and chlamydospores absent. Basidiospores ellipsoid, thin-walled, hyaline, smooth, IKI–, CB–, (5.0–)6.0 (–7.0) × (2.0–)3.0–3.5 μm, (ave = 6 × 3 μm), Q = 1.7–2.5, (ave = 2, n = 40).

Substrate: on fine woody debris.

Distribution: know from Brazilian and Chacoan subregions, in the Boreal Brazilian, Paraná, and southeastern dominions, including the Atlantic, Imer, and Xingu-Tapajós provinces (Fig 5A).

Specimens examined: BRAZIL, Amazonas, Panuré, Feb 1853, Spruce 195 (NY 730627, syntype of *Polyporus diabolicus*); collector unspecified, s.n. (NY 731050, type of *P*. *vernicosus*); Novo Airão, Parque Nacional de Anavilhanas, Igarapé Santo Antônio, 02º24'227''S, 60º58'215''W, 25 m elevation, on dead twig on the ground, 6 Dec 2013, ER. Drechsler-Santos DS1266 (FLOR 60361); Bahia, Wenceslau Guimarães, Estação Ecológica Wenceslau Guimarães, 18 Aug 2008, J. Pereira JAD3 (FLOR 60351); São Paulo, Iporanga, Parque Estadual Turístico do Alto Ribeira, Morro do Santana, 14 Dec 2014, G. Alves-Silva GAS679 (FLOR 60338); Pará, Belterra, Floresta Nacional de Tapajós, BR 163-KM 117, 03º21'213''S, 54º56'595''W, 29 Jan 2015, ER. Drechsler-Santos DS1695 (FLOR 60313).

Remarks: *Atroporus diabolicus* is characterized by the presence of strongly dextrinoid skeletal-binding "prickly" hyphae with a pointed apex in the trama of the tubes that arises above the hymenium, the rounded/truncate and sterile margin and the robust appearance of the basidiomata. *Atroporus dibolicus* is microscopically similar to *A*. *rufoatratus* and *A*. *infernalis*, however *A*. *rufoatratus* has tramal skeletal-binding hyphae developing a stalk that tend to be slightly longer and narrower (49−93 × 2−8 μm), with a rounded and projected apex (lacking spine-like short branches), similar to those of *A*. *infernalis*. Macrocopically, *A*. *infernalis* is hitherto known by a short and lateral stipe with pileus flat and flabelliform, *A*. *diabolicus* is central to eccentrically stipitate with pileus flat and circular, and *P*. *rufoatratus* centrally stipitate with pileus circular, depressed to slightly infundibuliform.

***Atroporus rufoatratus*** (Berk.) Palacio, Reck & Robledo comb. nov. (Figs 2J, 2J1, 2K, 2K1, 3F, 4C2, 7A and 7B).

**Fig 7:**
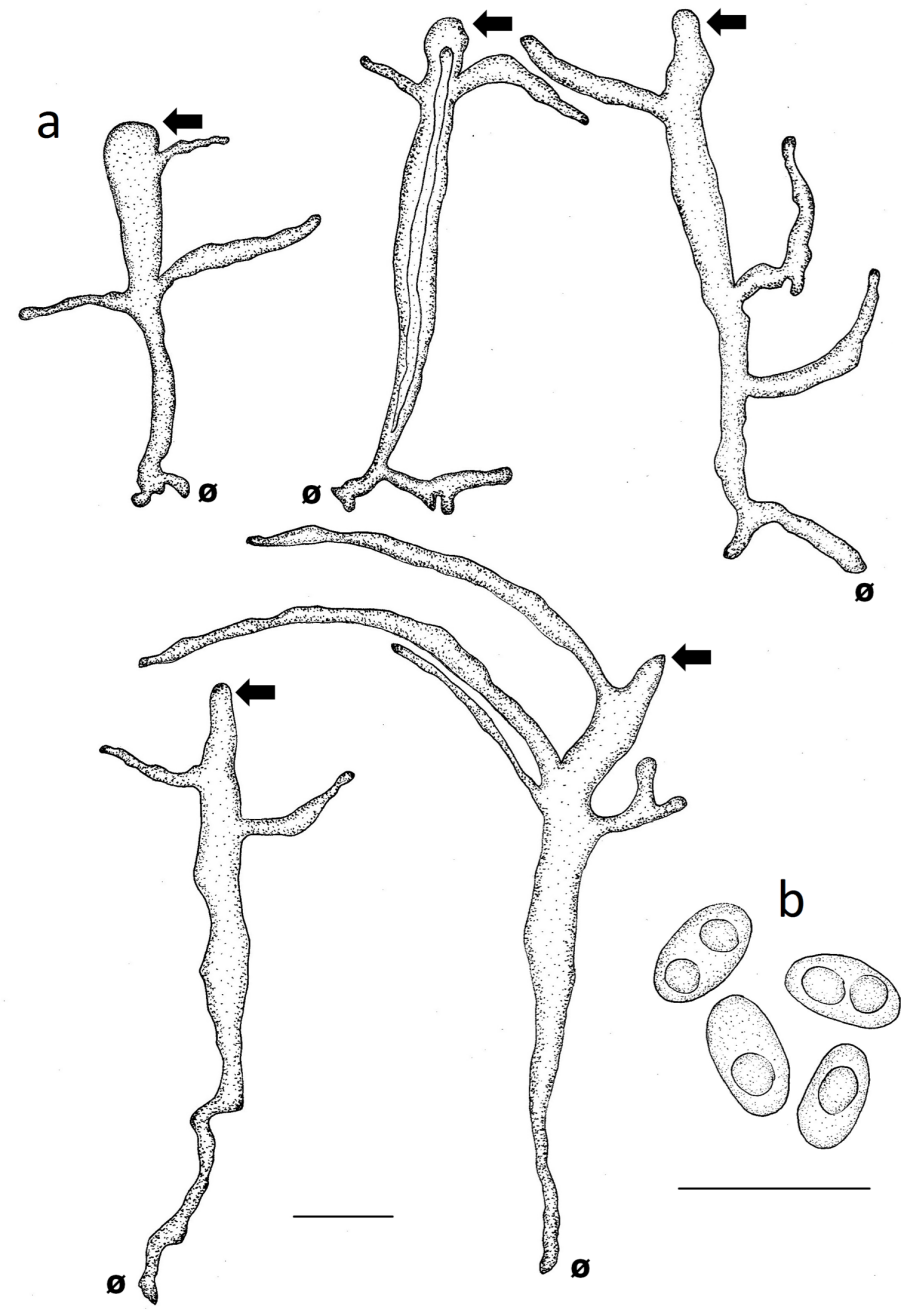
Microscopical features of *Atroporus rufoatratus*. **a**. tramal hyphae (LDA138). **b**. ellipsoid basidiospores (LDA139). Ø = clamp scar. Left arrow: Pointed apex of the hyphae. Scale bars = 10 μm.

MycoBank no.: MB 819626

≡ *Polyporus rufoatratus* Berk. Hooker's J. Bot. Kew Gard. Misc. 8: 174 (1856)!

Basidiomata annual, centrally stipitate, solitary; pileus circular, depressed to slightly infundibuliform, up to 2.6 (–4) cm in diameter and 1.5 mm thick; pilear surface dark reddish brown (2.5YR2.5/4), glabrous, radially striate; margin deflexed to inflexed, steril, with a black cuticle. Pore surface brownish yellow (10YR6/6); pores circular 4−7 per mm, 90−220(−250) μm (ave = 144.4 μm, n = 240/6); dissepiments entire to sligthly lacerate, (20−) 30−70(−90) μm thick, (ave = 49.2 μm, n = 240/6). Tubes concolorous with the context, not stratified, up to 0.8 mm long, decurrent to free. Context homogeneous, yellow (10YR7/6), up to 1 mm thick. Stipe cylindrical, solid, glabrous, smooth to slightly striate, bearing a black cuticle, up to 3.7 cm (−9.8 cm) cm long and 3 mm in diam. Hyphal system dimitic with generative hyphae and skeletal-binding hyphae. Generative hyphae with clamps, hyaline, thin-walled, 2−3 μm thick, IKI−, CB−; skeletal-binding hyphae of two types. Arboriform skeletal-binding hyphae present in the context and the stipe, up to 160 μm long, 2−4.5 μm wide, straight to geniculated, thin to thick-walled, branched, with a short unbranched stalk (30−45 μm), 5−7 branches (up to 210 μm long and 1−3 μm wide) with an alternating arrangement and shortened as approaching the trama of the tubes, hyaline to yellowish in KOH, water, and lactofenol, IKI-. In the trama of the tubes they differ in the second type of hyphae (Fig 7A), skeletal-binding hyphae with a wider main stalk (49−93 μm long) developed just after the clamp scar (2−3 μm wide) that is enlarged especially in the central portion (4−8 μm wide), between the middle and apical portion 2 to 5 branches arise (1−3 μm wide), up to 76 μm long, with dichotomous branches, thin to thick-walled, the hyphal apex is round and projected above hymenium, hyaline to yellowish in KOH, water, and lactofenol, strongly dextrinoid changing to dark brown. Pileipellis as an anamorph matrix, 20−28 μm thick, pale yellow to dark orange. Cystidioles subulate, 13−20 × 5−7 μm, with a basal clamp; basidia clavate, 4-sterigmate, with a basal clamp, 17−21 × 6−8 μm. Basidiospores narrowly ellipsoid to rarely subcylindrical, thin-walled, hyaline, smooth, IKI−, CB−, 5.0−7.0 × 3.0−4.0 μm, (ave = 5.8 × 3.3 μm), Q = 1.8−2.3, (ave = 1.9, n = 120/6).

Substrate: on fine woody debris.

Distribution: occurs in the Atlantic, Imerí, Pantepui, and Paraná Forest and provinces (Fig 5B).

Specimens examined: ARGENTINA, Misiones: Oberá, Campo Ramón, Centro de Investigación Antonia Ramos, 27°26' S, 54°55' W, 300−500 m elevation, Feb 2015, N. Gómez NG134 (FLOR 60373); 1 Dec 2011, E. Grassi MEX0138 (BAFC 52291). BRAZIL, Amazonas, Panuré, collector unspecified s.n. (NY 730938, type of *Polyporus rufoatratus*); Roraima, Caracaraí, Estrada Manaus-Caracaraí, Km 513, Ac. Novo Paraíso, 21 Nov 1977, I. Araujo 651 (NY 1972060); Km 328, 16 Nov 1977, I. Araujo 494 (NY 1972061); Km 360, 19 Nov 1977, I. Araujo s.n. (NY 1972065); Santa Catarina, Santo Amaro da Imperatriz, 21 Mar 2015, M. Palacio MP153 (FLOR 60355); Plaza Caldas da Imperatriz, Trilha da Cascata, 27 Feb 2014, L. Dalpaz LDA129 (FLOR 60347); LDA138 (FLOR 60348); LDA 139 (FLOR 60349); Florianópolis, Lagoa do Peri, 08 Jan 2014, J. Prata JP1 (FLOR 60345); 15 Feb 2014, ER. Drechsler-Santos DS1311 (FLOR 60312); Naufragados, 10 Jan 2014, J. Prata JP10 (FLOR 60346); 15 Mar 2014, L. Dalpaz LDA140 (FLOR 60350); 23 Feb 2016, M. Palacio MP158 (FLOR 60357); Unidade de Conservação Ambiental Desterro, 2 Jun 2012, ER. Drechsler-Santos DS816 (FLOR 60305).

Remarks: This species is well characterized by the narrowly ellipsoid to rarely subcylindrical basidiospores and the skeletal-binding hyphae of the trama, strongly dextrinoid, with a widened main stalk and a round apex projected above the hymenium; macroscopically, it is characterized by its centrally stipitate basidiomata, infundibuliform dark reddish brown pilei, and slender stipe. *Atroporus diabolicus* is a related species but it has tramal skeletal-binding hyphae with a slightly shorter and wider stalk (41–75 × 3–11 μm) and a pointed and "prickly" apex, besides a more robust appearance of the basidiomata.

#### Comments on other taxa related to *Atroporus*

*Atroporus infernalis* (Berk.) Ryvarden, Norw. Jl Bot. 20: 2 (1973)!

Pore surface brown (10YR5/3); pores circular (5−)6−7 per mm; dissepiments entire to sligthly lacerate, 20−50(−70) μm thick, (X = 32.8 μm, n = 40/1). Hyphal system dimitic. Generative hyphae thin-walled, hyaline, with clamp connections, up to 4 μm in diam. Skeletal-binding hyphae thick-walled to solid, branched, hyaline (similar to *A*. *rufoatratus*), dextrinoid, up to 6 μm in diam. Basidiospores not seen.

Remarks: the type specimen is damaged, only a pilear fragment of 2 cm remaining in the voucher specimen. Berkley [43] described *P*. *infernalis* based on a collection from Minas Gerais (Brazil) as an allied species of *P*. *varius* (Pers.) Fr. and *P*. *dictyopus*, but as a “very distinct species”. *Polyporus infernalis* was later transferred to *Atroporus* [16] based on the dextrinoid and modified skeletal-binding hyphae in the trama of the tubes. After our revision of the type we confirm the presence of this feature, endorsing that this species belongs to *Atroporus*; we also observed the sterile margin as mentioned in the protologue. *Atroporus infernalis* is related to *A*. *rufoatratus* but it differs in having a short and lateral stipe, and flabelliform pileus (protologue information [43]). Unfortunately, basidiospores were not able to be observed and the poor condition of the type did not allow us to compare it to the other specimens.

Specimen examined: Brazil. Minas Gerais: Arraial das Merces, Oct. 1840 (NY 730749, type of *Polyporus infernalis*).

*Neodictyopus* Palacio, Robledo, Reck & Drechsler-Santos gen. nov.

MycoBank no.: MB819629.

Etymology. *neo* (Lat.): new; *dictyopus* (Gre.): reticulate stipe surface of *Polyporus dictyopus* s.l.; the *new dictyopus*, in reference to the recognition of a new genera segregated from *P*. *dictyopus* complex.

Basidiomata annual, lateral to eccentrical, rarely centrally stipitate; pileus reniform to flabelliform; pilear surface glabrous, radially striate, dark reddish brown; margin irregular, wavy, and lobed to decurved and entire. Pores circular. Context homogenous, yellow to light brown. Stipe cylindrical, solid, reticulated to longitudinally striate, bearing a black cuticle (Fig 2A, 2B, 2C and 2D). Hyphal system dimitic; generative hyphae clamped, hyaline, thin-walled, branched skeletal-binding hyphae dominating, arboriform, hyaline, IKI− to slightly dextrinoid (only in mass) in the trama of the tubes. Basidia clavate, 4-sterigmate. Basidiospores subcylindrical to bacilliform, thin-walled, smooth, hyaline, IKI–(Figs 3B, 3D, 3G, 3H, 4D, 4E, 4F1, 4F2, 4F3 and 4F4).

Type species. *Neodictyopus atlanticae* Palacio, Robledo & Drechsler-Santos.

Remarks: *Neodictyopus* is characterized by its subcylindrical to bacilliform basidiospores, reniform to spatulate pileus, and skeletal-biding hyphae of arboriform type, slightly dextrinoid (when in mass) in the trama of the tubes. So far, the genus is neotropical (Fig 5C, 5D and 5E), but probably pantropical, since some specimens from the Paleotropics clustered together with the Neodictyopus clade. All the species grow on dead wood, typically dead fallen branches of relatively thin diameter (up to 10 cm diam) and produce white rot on the substrate. *Neodictyopus* is morpholically similar to other *Polyporus* species; however, *P*. *tuberaster*, the type species of *Polyporus*, has fleshy basidiomata when fresh, and pileus upper surface whitish to ochraceous covered with scales. Macroscopically, *Neodictyopus* is more similar to *Atroporus*, but the ellipsoid to subcylindrical basidiospores and strongly dextrinoid skeletal-biding hyphae from the trama of the tubes are unique to latter.

***Neodictyopus atlanticae*** Palacio, Robledo & Drechsler-Santos sp. nov. (Figs 2A, 2A1, 3H, 4F4, 8A and 8B).

**Fig 8:**
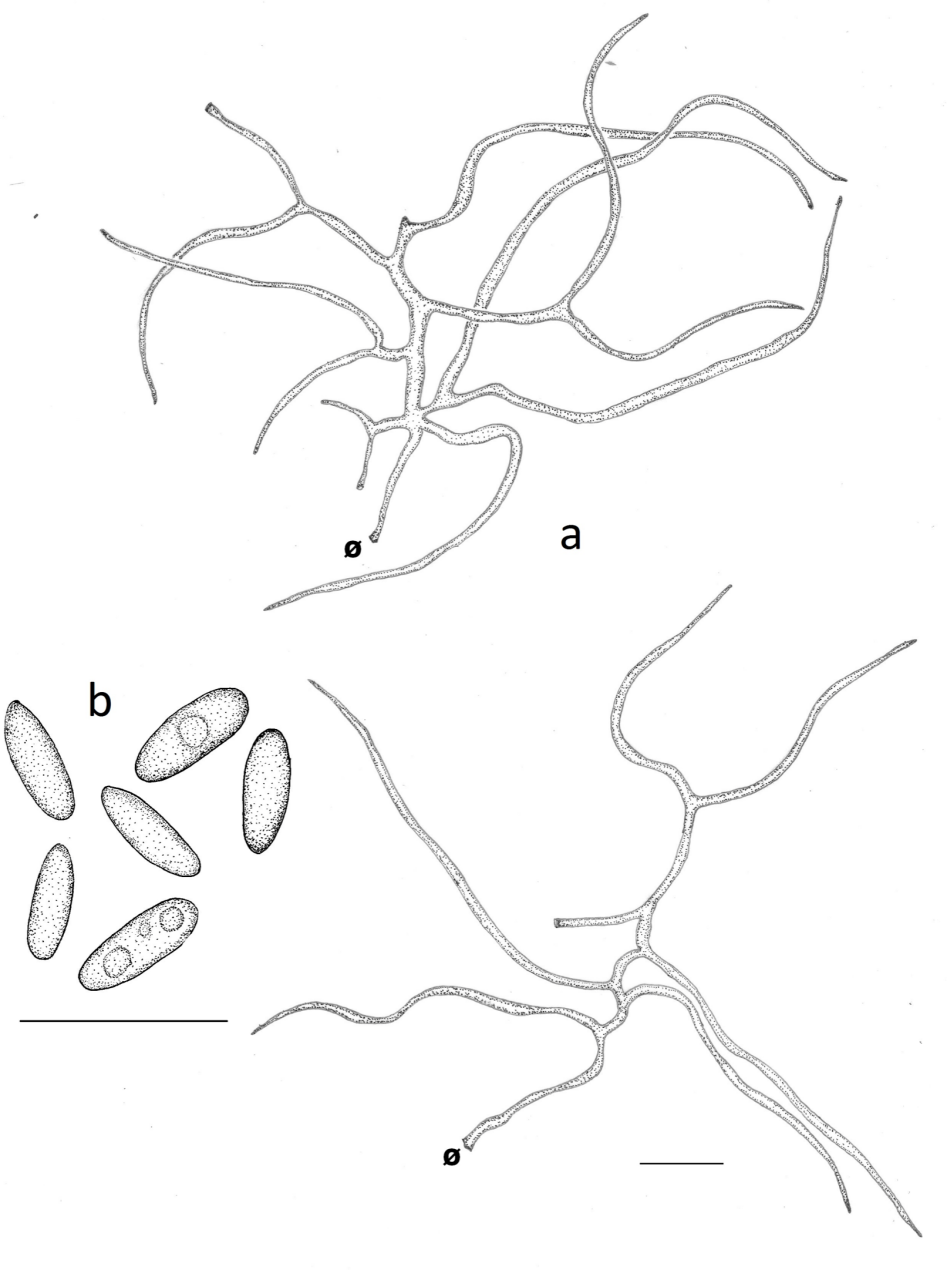
Microscopical features of *Neodictyopus atlanticae*. **a**. tramal hyphae (DS1284). **b**. cylindrical basidiospores (FB351). Ø = clamp scar. Scale bars = 10 μm.

MycoBank no.: MB819631.

Holotype: Brazil, Santa Catarina, Santo Amaro de Imperatriz, Caldas da Imperatriz, 15 Nov 2013, ER. Drechsler-Santos DS1285 (FLOR 60309).

Etymology: atlanticae (Latin) Atlantic, referring to the species distribution, known to the Atlantic province.

Basidiomata annual, laterally to eccentrically stipitate, tipically gregariuos, up to four basidiomata in 10 cm of wood; pileus reniform, up to 4.1 cm in diameter and 1.5 mm thick; pilear surface strong brown (7.5YR5/8) to dark reddish brown (2.5YR2.5/4), radially striate, glabrous; margin irregular, wavy and lobed. Pore surface brownish yellow (10YR6/8) to grayish brown (10YR5/2); pores circular 5−9 per mm, (80−)90−170(−180) μm (ave = 121.3 μm, n = 160/4); dissepiments entire to slightly lacerated, 20−90(−100) μm thick, (ave = 48.3 μm, n = 160/4). Tubes concolorous with the pore surface, not stratified, up to 0.5 mm long, decurrent and irregularly attached to the stipe. Context homogeneous, light brown (7.5YR6/4), up to 1 mm thick. Stipe cylindrical, solid, slender, glabrous, longitudinally striated, bearing a black cuticle, up to 2 cm long and 2 mm in diam. Hyphal system dimitic with generative hyphae and skeletal-binding hyphae. Generative hyphae with clamps, hyaline, thin-walled, 1−2.5 μm thick, IKI–, CB–, more easily observed in the tubes. Skeletal-binding hyphae hyaline to yellowish in KOH or water, nondextrinoid to ocasionally weakly dextrinoid, CB–. Stipe, context and trama of the tubes composed mainly of skeletal-binding hyphae with a loose arboriform branching pattern (Figs 3B and 4D), up to 350 μm long, 2.5−5 μm wide, thick-walled, geniculated, with a short unbranched stalk (20−90 μm) and then with 2−5 branches (up to 550 μm long) with an alternating arrangement. Skeletal-binding hyphae in the trama (Fig 4E) shorter (80−150 μm) than those at stipe and context, becoming shorter (up to 90 μm) approaching the dissepiments with more (5−9) and shorter ramifications (Figs 3D and 8A). Cystidiole subulate, 14−20 × 4−5 μm, with a basal clamp. Basidia clavate, 4-sterigmate, 19−21 × 5−6 um. Basidiospore cylindrical, thin-walled, hyaline, smooth, IKI−, CB−, (6.0−)6.5−8.0 × 2.0−3.0 μm, (ave = 6.3 × 2.1 μm), Q = 2−3.5, (ave = 3, n = 160/4).

Substrate: on fine woody debris.

Distribution: only known from the Atlantic province in the Paraná dominion (Fig 5E).

Specimens examined: BRAZIL, Santa Catarina, Blumenau, Parque Nacional da Serra do Itajaí, Trilha da Chuva, 27°03'073'' S, 49°04'5320'' W, 17 Jan 2015, F. Bittencourt FB351 (FLOR 60372); Santo Amaro da Imperatriz, Caldas da Imperatriz, Hotel Caldas da Imperatriz; 15 Nov 2013, ER. Drechsler-Santos DS1284 (FLOR 60308); DS1286 (FLOR 60310).

Remarks: *Neodictyopus atlanticae* is well characterized by lateral to eccentrically stipitate basidiomata, well developed and slender stipe, reniform pileus with irregular, wavy and lobed margin, and the gregarious habit.

***Neodictyopus dictyopus*** (Mont.) Palacio, Robledo & Drechsler-Santos comb. nov. (Figs 2B, 2B1, 2C, 4F1, 4F2, 9A, 9B and 9B1).

**Fig 9:**
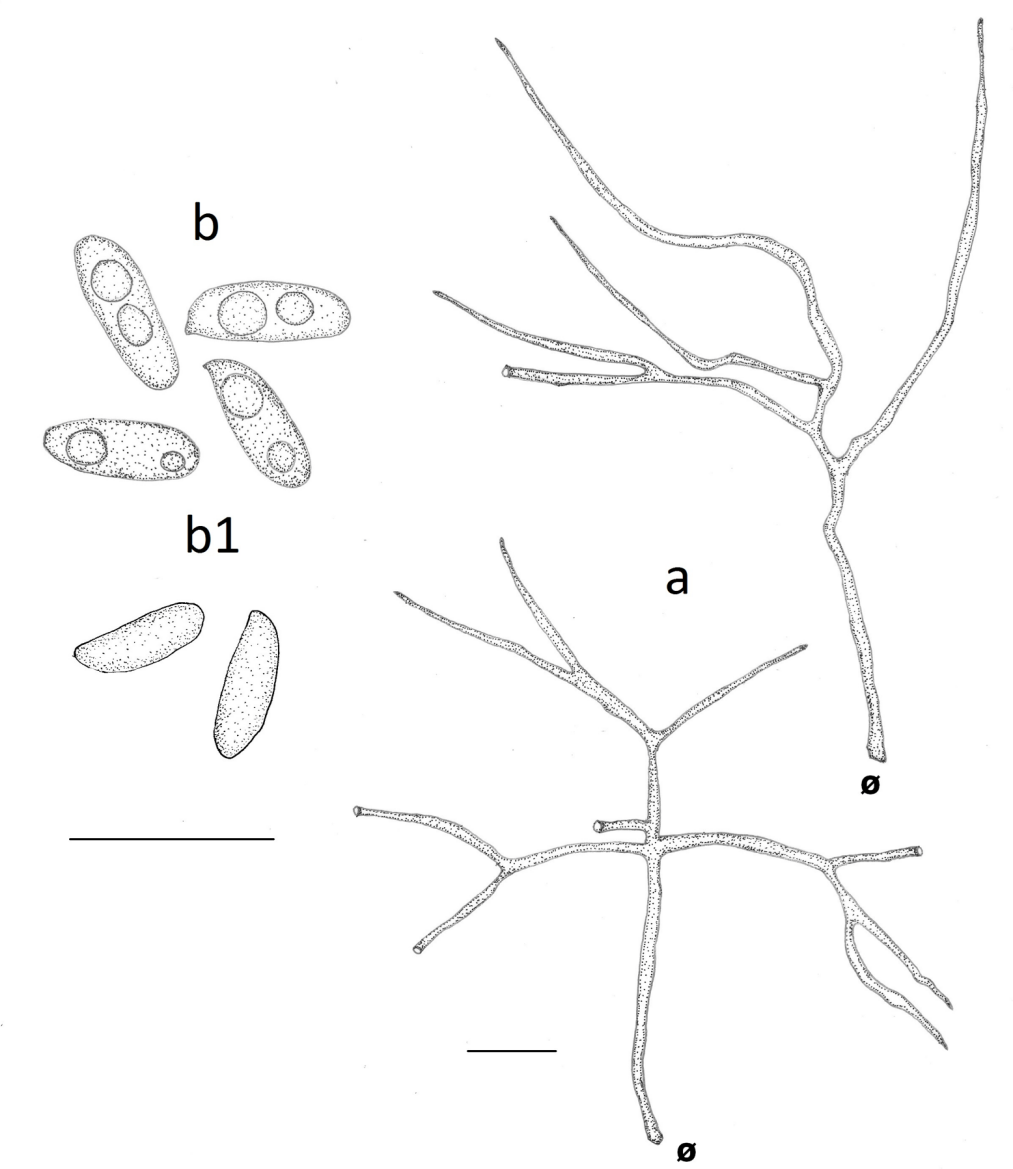
Microscopical features of *Neodictyopus dictyopus*. **a**. tramal hyphae. **b**. cylindrical basidiospores (GAS281). **b1**. cylindrical basidiospores (BPI US207664, type of *N*. *dictyopus*). Ø = clamp scar. Scale bars = 10 μm.

MycoBank no.: MB819633.

Basionym: *Polyporus dictyopus* Mont. Annls Sci. Nat., Bot., sér. 2 3: 349 (1835)!

Basidiomata annual, laterally stipitate, solitary to clustered; pileus round, reniform to flabeliform, up to 7.5 cm in diameter and 2.5 mm thick; pilear surface dark reddish brown (5YR3/2) to yellowish red (5YR5/8), radially striate, glabrous; margin irregular, wavy and lobed. Pore surface brown (10YR5/3); pores circular 6−9 per mm, 90−130(−150) μm, (ave = 108.3 μm, n = 120/3); dissepiments entire to sligthly lacerate 20−70(−80)μm thick, (ave = 36.7 μm, n = 120/3). Tubes concolorous with the pore surface, not stratified, up to 0.8 mm long, decurrent and irregularly attached to the stipe. Context homogeneous, yellow (10RY7/8), up to 1 mm thick. Stipe cylindrical, solid, glabrous, reticulated, bearing a black cuticle, short up to 1.5 cm long and 8 mm in diam. Hyphal system dimitic with generative hyphae and skeletal-binding hyphae. Generative hyphae with clamps, hyaline, thin-walled, 1−3 μm thick, IKI–, CB–, more easily observed in the tubes. Skeletal-binding hyphae hyaline to yellow in KOH and water, IKI- to occasionally weakly dextrinoid, CB–. Stipe, context and trama of the tubes composed mainly by skeletal-binding hyphae with a loose arboriform branching pattern, up to 250 μm long, 2.5−5 μm wide, thick-walled, geniculated, with a short unbranched stalk (25−75 μm) and then with 2−5 branches (up to 250 μm long) with an alternating arrangement. In the trama, the skeletal-binding hyphae are shorter (up to 107 μm) than those of stipe and context, and even shorter (up to 84 μm) as approaching the dissepiments where there are more branched (4−7) and shorter (up to 85 μm long) (Fig 9A). Cystidioles subulate, 17−21 × 4−5 μm, with a basal clamp. Basidia clavate, 4-sterigmate, 15−21 × 5−7 um. Basidiospores subcylindrical to rarely narrowly cylindrical, thin-walled, hyaline, smooth, IKI–, CB–, (6.0−)6.5−8.0 × 2.0−3.0 μm, (ave = 7 × 2.6 μm), Q = 2.5−3.3 (ave = 2.81, n = 120/3).

Substrate: on fine woody debris.

Distribution: This species was originally described from temperate forest of the Juan Fernández archipelago (Chile), being currently and also found in the Cerrado province of Mato Grosso state (Brazil) (Fig 5D).

Specimens examined: BRAZIL, Mato Grosso, Cuiabá, Chapada dos Guimarães, Parque Nacional da Chapada dos Guimarães, 15°24'28.3"S, 55°50'00.3"W, 27 Nov 2011, G. Alves-Silva GAS60 (FLOR 60365); 15°24'30.0"S, 55°49'57.5"W, 05 Aug 2012, G. Alves-Silva GAS272 (FLOR 60366); GAS281 (FLOR 60367); Véu da Noiva, 15°24’25”S, 55°50’17”W, 19 Jun 2011, V. Ferreira-Lopes VFL18 (FLOR 60364). CHILE, Juan Fernandez, Bertero 1683 (BPI 207664, holotype of *P*. *dictyopus*).

Remarks: *Neodictyopus dictyopus* is characterized by having basidioma laterally stipitate, with short, robust, black, and reticulated stipe, margin irregular, wavy and lobed, variable pilear surface color, and subcylindrical to rarely narrowly cylindrical basidiospores. The Brazilian specimens examined for this study are linked to the type specimen by morphological comparison, despite the disjunct distribution. In order to better define the circumscription and distribution of *N*. *dictyopus*, more collections from the type locality are needed. *Neodictyopus dictyopus* can be differentiated from *N*. *atlanticae* by the short, robust, and lateral stipe, and the smaller basidiospores.

***Neodictyopus gugliottae*** Palacio, Grassi & Robledo sp. nov. (Figs 2D, 2D1, 3G, 4F3, 10A and 10B).

**Fig 10:**
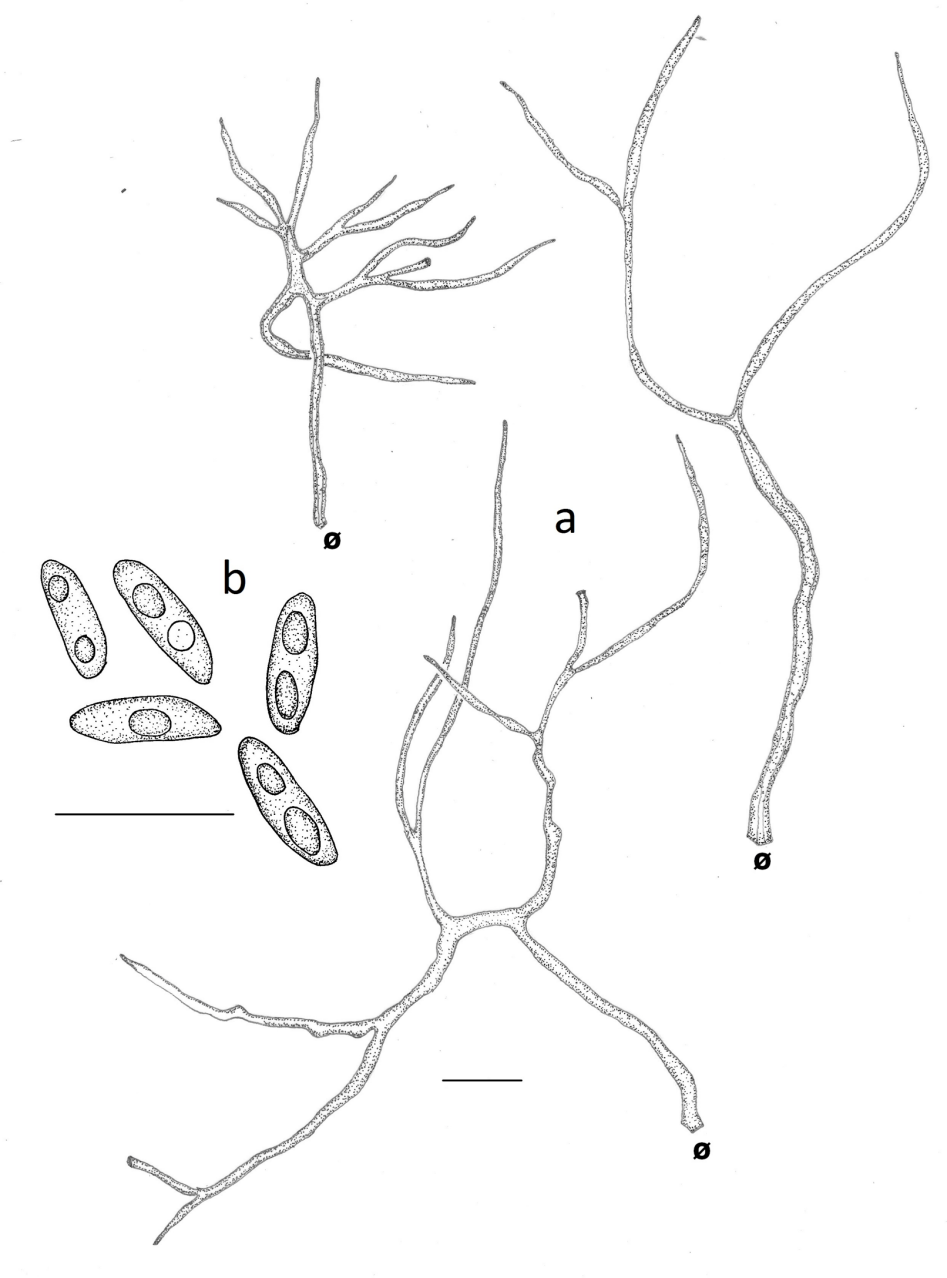
Microscopical features of *Neodictyopus gugliottae*. **a**. tramal hyphae. **b**. cylindrical basidiospores (GAS622). Ø = clamp scar. Scale bars = 10 μm.

MycoBank no.: MB819632.

Holotype: Brazil, Santa Catarina, Joaçaba, Parque Ecológico Municipal Rio do Peixe, 27 Sep 2014, G. Alves-Silva GAS622, (FLOR 60333).

Etymology: in honor of Dr. Adriana Gugliotta, a Brazilian expert in polypores, for its contributions to our knowledge of polypore fungi diversity.

Basidiomata annual, laterally stipitate, solitary; pileus flabelliform to slightly spathulate, up to 1.5 cm in diameter and 2 mm thick; pilear surface reddish brown (2.5YR4/4) to dark reddish brown (2.5YR2.5/4), radially striate, glabrous; margin decurved and entire. Pore surface yellow (10YR7/6); pores circular to slightly radially elongated (5−)6−7 per mm, (120−) 129–190(−200) μm, (ave = 161.8, n = 40); dissepiments entire, (20−)30−60(−70) μm thick, (ave = 44.8 μm, n = 40/1). Tubes concolorous with the pore surface, not stratified, up to 1 mm long. Context homogeneous, yellow (10YR8/8), up to 1 mm thick. Stipe cylindrical, solid, slender, longitudinally striate, glabrous, bearing a black cuticle, up to 2.3 cm long and 2 mm in diam. Hyphal system dimitic with generative hyphae and skeletal-binding hyphae. Generative hyphae with clamps, hyaline, thin-walled, 2−3 μm thick, IKI–, CB–, more easily observed in the tubes. Skeletal-binding hyphae hyaline to yellowish in KOH and water, IKI–, CB–. Stipe, context and trama of the tubes composed mainly of skeletal-binding hyphae with a loose arboriform branching pattern, up to 310 μm long, 3−5 μm wide, thick-walled, geniculated, with a short unbranched stalk (92−155 μm) and then with 2−4 branches (up to 190 μm long) with an alternating arrangement. Skeletal-binding hyphae from the tubes are shorter (up to 120 μm) than in stipe and context, and become shorter (up to 90 μm) approaching the dissepiments where there are more (3−6) and shorter ramifications (Fig 10A). Cystidiole subulate, 12−15 × 3−5 μm, with a basal clamp. Basidia clavate, 4-sterigmate, 21−23 × 5−6 um. Basidiospores subcylindrical, narrowly cylindrical to bacilliform, thin-walled, hyaline, smooth, IKI−, CB−, 6.0−9.0 × 2.0−2.5 μm, (ave = 7.6 × 2.1 μm), Q = 2.8−4.5, (ave = 3.6, n = 40/1).

Substrate: on fine woody debris.

Distribution: only known from Araucaria and Paraná Forest provinces in Brazil and NE Argentina (Fig 5C).

Specimens examined: ARGENTINA, Misiones, Oberá, Campo Ramon, Centro de Investigación Antonia Ramos, 27°26' S, 54°55' W, 300−500 m elevation, 10 Dec 2011, E. Grassi CI110 (BAFC 52641).

Remarks: *Neodictyopus gugliotae* is characterized by subcylindrical to bacilliform basidiospores, the eccentrically stipitate basidiomata with a circular pileus. *Neodictyopus atlanticae* has a similar slender and developed stipe but it differs in its irregular, wavy and lobed pileus margin and shorter basidiospores.

#### Comments on taxa related to *Neodictyopus*

*Polyporus blanchetianus* Berk. & Mont., Annls Sci. Nat., Bot., sér. 3 11: 238 (1849)!

Pore surface brown (10YR5/3); pores circular 6−7 (−8) per mm; dissepiments entire, (20−)30−50(−60) μm thick, (ave = 41.9 μm, n = 40/1). Hyphal system dimitic. Generative hyphae thin-walled, hyaline, with clamp connections, up to 5 μm in diam. Skeletal-binding hyphae from the tubes thick-walled to solid, branched, hyaline, IKI−, up to 5 μm in diam. Basidiospores subcylindrical to bacilliform, thin-walled, hyaline, smooth, IKI−, CB−, 6.0–6.5 × 2.0 μm Q = 2.8−4.5, (ave = 3.6, n = 20/1).

Remarks: Type specimen damaged, only a pilear fragment remaining in the voucher specimen. Based on the cylindrical basidiospores and skeletal-binding hyphae IKI-, it is possible to recognize *P*. *blanchetianus* as a *Neodictyopus* member; however, given the poor condition of the holotype, we prefer to consider *P*. *blanchetianus* as a dubious species. Additional specimens from the type locality, Salvador (Bahia) according to Góes-Neto [44], are needed to confirm its correct placement.

Specimen examined: Brazil, Bahia, Salvador, Blanchet s.n. (NY 730532, holotype of *Polyporus blanchetianus*).

#### Other species possibly included in the genus *Atroporus* and *Neodictyopus*

Despite the fact that we could not examine the types of the species cited below, protologues present information that suggests that all taxa, commonly treated as synonyms of *P*. *dictyopus*, might be distinct species and should be kept as *insertae sedis* until the appropriate revision:

*Fomes holomelanus* Berk. ex Cooke, Grevillea 15(no. 74): 51 (1886).

*Melanopus scabellus* Pat., Bull. Soc. mycol. Fr. 16: 178 (1901).

*Polyporus atroumbrinus* Berk., Hooker´s J. Bot. Kew Gard. Misc. 8: 199 (1856).

*P*. *nephridis* Berk., Hooker's J. Bot. Kew Gard. Misc. 8: 195 (1856)

*P*. *parvimarginatus* Speg., Anal. Soc. Cient. Argent. 16(6): 280 (1883).

*P*. *rhizomorphus* Mont., Annls Sci. Nat., Bot., sér. 2 13: 202 (1840).

*Polystictus puiggarii* Speg., Boln Acad. Nac. Cienc. Córdoba 11(4): 441 (1889).

## Discussion

Inferences from previous phylogenetic studies including specimens identified as *P*. *dictyopus* have been limited by the small number of sequences and did not link the results with morphological studies [7,8,10]. Our reconstructions revealed that *P*. *dictyopus*, as it is currently understood [1,2,15], is polyphyletic. Moreover, it includes species belonging to two well defined lineages, both presenting distinct morphological features, supporting them as distinct genera, *Atroporus* and *Neodictyopus*.

*Atroporus* conforms a strongly supported clade in both BI and MP analyses (Fig 1) and includes *A*. *diabolicus*, the generic type species, and *A*. *rufoatratus*. Within *Atroporus*, two highly supported lineages (Fig 1) represent 2 species: *A*. *diabolicus* represented by one specimen from the Imerí province (Amazonas, Brazil), and *A*. *rufoatratus* represented by three specimens from the Atlantic province (Santa Catarina, Brazil). Both species have ellipsoid to rarely cylindrical basidiospores (Q = 1−2), strongly dextrinoid skeletal-binding hyphae from the trama of tubes, protruding the hymenium with an acute apex, and centrally to eccentrically stipitate basidiomata.

*Neodictyopus* is strongly supported by both BI and MP analyses (Fig 1) and encompasses four lineages. *N*. *atlanticae* is represented by a strongly supported clade with three specimens from the Atlantic province (Santa Catarina, Brazil), the type species of the genera. The clade of *N*. *dictyopus* is composed by three specimens from the Cerrado province (Mato Grosso, Brazil). *Neodictyopus gugliottae* is represented by a strongly supported clade formed by two specimens from Araucaria and Paraná Forest provinces (São Paulo, Brazil and Misiones, Argentina), respectively. Finally, there is a paleotropical clade (BPP = 1.00, BS = 100%) with three samples from subtropical Asia, which are not taxonomically treated in this work. *Neodictyopus atlanticae*, *N*. *dictyopus*, and *N*. *gugliottae* share cylindrical basidiospores, reniform pileus, and a lateral to occasionally eccentrical stipe.

The study of the type specimen of *P*. *dictyopus* showed morphological similarities with the Cerrado specimens. The reticulated stipe surface (Fig 2B1), the short (up to 1.5 cm) and wide (up to 8 mm) stipe, and the flabeliform pileus are macromorphologically similar features. Micromorphologically, basidiospore shape and size (Fig 9B and 9B1) and skeletal-binding hyphae with a loose arboriform branching pattern and weakly dextrinoid reaction in the dissepiments are identical. Molecular data from the *N*. *dictyopus* type or from other type locality specimens were not possible to be used in our study. However, based on the morphological similarities, our specimens (GAS60; GAS272; GAS281, VFL18) were assumed as conspecific.

*Atroporus* and *Neodictyopus* share similar hyphal system in the context of pileus and stipe, with generative hyphae with clamps and dominant skeletal-binding hyphae. Both genera have basidiomata with a dark reddish brown cuticle on the pilear surface, becoming even blackish in *A*. *diabolicus*, and have a substrate preference for fine woody debris (diameter 5–9 cm). *Atroporus* species can be differentiated by its ellipsoid basidiospores, strongly dextrinoid skeletal-binding hyphae in the trama of tubes with projected apex, and centrally to eccentrically stipitate basidiomata. Differently, *Neodictyopus* species have cylindrical basidiospores, nondextrinoid to weakly dextrinoid (only in mass) skeletal-binding hyphae, and lateral to eccentric stipitate basidiomata.

The distinct skeletal-binding hyphae of the trama, here treated as typical for *Atroporus*, were once considered as cystidia [16] and/or as modified skeletal-binding hyphae for the *P*. *dictyopus s*.*l*. [1, 15–17]. Meticulous examinations of the hyphal system according Decock et al. [24] allow us to observe and describe whole hyphae, and then reinterpret as a unique type of skeletal-binding hyphae characteristic of *Atroporus*.

In this study, *Neodictyopus* was recovered as a sister group of *Picipes*. Our results also bring new phylogenetic information about *Atroporus*, which appears as a sister clade of the remaining *Neodictyopus* and *Picipes*. These three genera formed a strongly supported clade (BPP = 1.00, BS = 98%, Fig 1), in which all the species share the black cuticle in the stipe, the principal character that defines *Melanopus sensu* Patouillard and Melanopus group *sensu* Núñez & Ryvarden [1]. However, other species (e.g. *P*. *leprieurii*, *P*. *guianensis*, and *P*. *varius*), that present the same cuticle, are not related to those clades, reinforcing Melanopus group is an artificial group as previously pointed out [10].

*Atroporus* can be easily differentiated from *Picipes* and *Neodictyopus* by its mainly ellipsoid basidiospores and strongly dextrinoid skeletal-binding hyphae from the tubes with protruding apex. *Neodictyopus* and *Picipes* are differentiated by their distribution; apparently *Picipes* [*Pi*. *badius* (Pers.) Zmitr. & Kovalenko, *Pi*. *melanopus* (Pers.) Zmitr. & Kovalenko, and *Pi*. *tubaeformis* (P. Karst.) Zmitr. & Kovalenko] is restricted to temperate zones in North hemisphere, whereas *Neodictyopus* could be restricted to Tropical and Subtropical regions.

*Polyporus austroandinus* (Pers.) Fr. is another related species that also has basidiomata with a stipe bearing a black cuticle, similar to *Neodictyopus* species. Nevertheless, the species has larger pores (4–5 per mm) and basidiospores [(–8)9–11.5 × 3–3.8(–4)], and grows in the temperate zones of the southern Andes forest [10].

Other white rot polypore genera that share characters with *Atroporus* and *Neodictyopus* can easily be morphologically differentiated. *Lentinus* and *Panus* Fr. also have stipitate basidiomata, a dimitic hyphal system, and cylindrical to subellipsoid, smooth, and inamyloid basidiospores [11,45], but present gilled basidiomata. *Pseudofavolus* Pat. also produces stipitate and poroid basidiomata, and has a similar hyphal system, however the larger basidiospores (more than 10 um), the gelatinous subhymenium and the presence of dendrohyphidia differentiated this genus [1] (Núñez & Ryvarden). *Datronia* share similar microscopic characters, but present effused-reflexed basidiomata with dendrohyphidia [46]. *Echinochaete* has a dimitic hyphal system similar to *Atroporus*, with dextrinoid arboriform skeletal-binding hyphae and generative hyphae with clamps, however the former has spinulose setoid elements on the pilear surface and in the hymenium [47].

The reexamination of morphological groups and species complexes within *Polyporus* is required in order to classify the genus in a less artificial way. Independent inspection of the hyphal system from the trama of the tubes, context, and pileus, the dextrinoidity of the structures, as well as basidiospore shape comparison (from the Q value), could assist the detection of morphological patterns within clades already recognized as the Melanopus clade sensu Dai et al. [10].

### Key to the species of *Atroporus* and *Neodictyopus*

1 Basidiospores usually ellipsoid, skeletal-binding hyphae from the tubes strongly dextrinoid with a well differentiated and protruding apex protruding into the hymenium                *Atroporus* 2
1 Basidiospores subcylindrical to bacilliform, skeletal-binding hyphae from the tubes IKI− to occasionally weakly dextrinoid, without differentiated apex                *Neodictyopus* 3
2 Skeletal-binding hyphae from the tubes with a sharply spinose pointed apex, basidiomata robust, generally with 2−3 tube layers, sometimes in old specimens with a black cuticle covering the hymenophore, stipe robust (up to 3.2 cm long × 0.5 cm diam.)                *A*. *diabolicus*
2 Skeletal-binding hyphae from the tubes with a smooth rounded apex, basidiomata slender, always with one tube layer, stipe slender (up to 9.8 cm long × 0.3 cm diam.)                *A*. *rufoatratus*
3 Pilear margin regular, decurved, and entire, basidiospores subcylindrical to bacilliform, Q = 2.8−4.5                *N*. *gugliottae*
3 Pilear margin irregular, wavy, and lobed, basidiospores subcylindrical to narrowly cylindrical, Q = 2−3.5                4
4 Basidiomata eccentrically stipitate, stipe perpendicular to the pileus (aprox. 90°), slender (up to 2 mm in diameter), up to 2 cm long, pileus reniform, basidiospores subcylindrical to rarely narrowly cylindrical, Q = 2−3.5                *N*. *atlanticae*
4 Basidiomata laterally stipitate, stipe horizontal to the pileus (aprox. 180°), robust (up to 10 mm), up to 1.5 cm long, pileus reniform to flabelliform, basidiospores subcylindrical to rarely narrowly cylindrical, Q = 2.5−3.3                *N*. *dictyopus*

## Supporting information

S1 File nrITS, nrLSU, and RPB2 data set final alignment. (FAS)

S2 File nrITS and nrLSU data set final alignment. (FAS)
